# Analysis of Circulating and Urinary Levels of hsa-miRNA-770-5p in Diabetic Nephropathy

**DOI:** 10.3390/biom16040545

**Published:** 2026-04-08

**Authors:** Dimitar Nikolov, Georgi Nikolov, Mariela Geneva-Popova, Stanislava Popova-Belova, Mladen Naydenov, Mari Georgieva Karusheva

**Affiliations:** 1 Second Department of Internal Diseases, Faculty of Medicine, Clinic of Nephrology, University Hospital “St. George”, Medical University—Plovdiv, 4002 Plovdiv, Bulgaria; dimitar.nikolov@mu-plovdiv.bg (D.N.); georgi.nikolov@mu-plovdiv.bg (G.N.); 2 Department of Propedeutics of Internal Diseases “Prof Anton Mitov”, Faculty of Medicine, Clinic of Rheumatology, University Hospital “St. George”, Medical University—Plovdiv, 4002 Plovdiv, Bulgaria; spopova92@abv.bg (S.P.-B.); marikarusheva1@gmail.com (M.G.K.); 3 Department of Human Anatomy and Physiology, Faculty of Biology, University of Plovdiv, 4000 Plovdiv, Bulgaria; mldlefrancais@abv.bg

**Keywords:** *miRNA-770-5p*, diabetic nephropathy, type 2 diabetes mellitus, biomarker, serum miRNA, urinary miRNA levels

## Abstract

Background: Diabetic nephropathy (DN), also referred to as diabetic kidney disease, represents one of the most common microvascular complications of type 2 diabetes mellitus (T2DM) and remains a leading cause of end-stage renal disease worldwide. Conventional clinical markers, including albuminuria and estimated glomerular filtration rate (eGFR), are widely used for diagnosis and staging but may have limited sensitivity for detecting early renal injury and predicting disease progression. In recent years, circulating *microRNAs (miRNAs)* have emerged as promising non-invasive biomarkers that reflect underlying molecular mechanisms of diabetic nephropathy and may complement traditional clinical indicators. Objective: The present study aimed to evaluate serum and urinary levels of *hsa-miRNA-770-5p* across different stages of diabetic nephropathy and to assess its potential diagnostic value in relation to established indicators of renal function. Methods: A total of 257 participants were included and divided into four groups: healthy controls, patients with T2DM without nephropathy, patients with T2DM and DN in CKD stages I–II, and patients with DN undergoing maintenance hemodialysis (MHD). Serum and urinary levels of *miRNA-770-5p* were measured using quantitative real-time polymerase chain reaction (qPCR) and analyzed using the 2^−ΔΔCt^ method. Statistical analyses included comparisons between groups using ANOVA, correlation analyses with renal function parameters such as eGFR and proteinuria/albuminuria, and receiver operating characteristic (ROC) curve analysis to evaluate diagnostic performance. Results: Serum levels of *miRNA-770-5p* were significantly elevated in patients with DN and in patients undergoing maintenance hemodialysis compared with healthy controls and patients with T2DM without nephropathy. In contrast, urinary levels of *miRNA-770-5p* were markedly reduced in patients with DN. Serum levels in patients with T2DM without nephropathy were slightly lower than those observed in healthy controls. Significant correlations were identified between *miRNA-770-5p* levels and renal function parameters, including eGFR and proteinuria/albuminuria, supporting the biological relevance of this *microRNA* in renal injury. ROC curve analysis demonstrated good discriminatory ability for differentiating DN from T2DM without nephropathy (serum AUC = 0.82; urine AUC = 0.79). Conclusions: *hsa-miRNA-770-5p* demonstrates distinct and opposite patterns in serum and urine that correlate with the severity of diabetic nephropathy. The complementary changes observed in circulating and urinary levels support the potential of *miRNA-770-5p* as a non-invasive biomarker that may complement conventional clinical markers and provide additional insight into the molecular mechanisms involved in the development and progression of diabetic nephropathy.

## 1. Introduction

Diabetic nephropathy (DN), currently referred to as diabetic kidney disease, remains one of the most common microvascular complications of type 2 diabetes mellitus (T2DM) and represents the leading cause of end-stage renal disease worldwide, as emphasized in reports from the American Diabetes Association and the Kidney Disease: Improving Global Outcomes (KDIGO) organization [1,2]. Diagnosis and staging of DN are primarily based on albuminuria and estimated glomerular filtration rate (eGFR) [3]. However, these clinical indicators may not adequately reflect early molecular injury and may fail to identify patients with non-albuminuric phenotypes of diabetic kidney disease or to predict disease progression with sufficient accuracy [1,2,3,4]. These limitations highlight the need for sensitive molecular biomarkers capable of detecting subclinical renal damage and improving early risk stratification beyond conventional clinical parameters.

*MicroRNAs* (*miRNAs*) are small non-coding RNA molecules that regulate gene expression at the post-transcriptional level and are increasingly recognized as key regulators of pathways involved in diabetic nephropathy, including apoptosis, inflammation, oxidative stress, and fibrotic remodeling [1,2,3,4]. Due to their remarkable stability in biological fluids such as serum and urine and the feasibility of reliable quantitative detection, *miRNAs* have emerged as promising non-invasive biomarkers in chronic kidney disease [5,6,7,8,9]. Several studies have demonstrated altered circulating and urinary miRNA profiles in DN, with specific *miRNAs* correlating with renal function decline, albuminuria, and progression to end-stage renal disease [1,9]. These findings suggest that *miRNAs* may provide complementary information to established clinical markers such as albuminuria and eGFR. Accumulating evidence underscores the contribution of specific microRNAs to the pathophysiology of diabetic nephropathy. In particular, miR-21 has been implicated in renal fibrogenesis, and its therapeutic silencing has been shown to attenuate diabetic kidney disease in experimental models [10]. Moreover, circulating exosomal *microRNAs* have been associated with albuminuria and are increasingly recognized as potential indicators of early renal injury in diabetic nephropathy [11]. In the broader context of type 2 diabetes mellitus, circulating *microRNAs* have emerged as promising biomarkers, although their clinical interpretation may be influenced by factors such as sex-related differences [12]. In parallel, urinary *microRNAs* have been extensively investigated as non-invasive biomarkers reflecting intrarenal pathological processes in chronic kidney disease [13].

Among *miRNAs* implicated in diabetic complications, *hsa-miRNA-770-5p* has recently attracted attention because of its potential involvement in metabolic stress responses and apoptosis regulation. Wang et al. [2] reported increased levels of *miRNA-770-5p* in patients with T2DM and demonstrated its association with pancreatic β-cell dysfunction. Furthermore, Zhang et al. [3] showed that *miRNA-770-5p* promotes podocyte apoptosis under hyperglycemic conditions by targeting TP53-regulated inhibitor of apoptosis 1 (TRIAP1), suggesting a possible mechanistic link with glomerular injury. Despite these observations, data regarding circulating and urinary levels of *miRNA-770-5p* across different stages of diabetic nephropathy remain limited, and its relationship with renal functional impairment has not been sufficiently explored.

Recent literature highlights the growing importance of urinary *microRNAs* as non-invasive biomarkers in diabetic kidney disease, with systematic reviews demonstrating their potential diagnostic and prognostic value in reflecting renal injury and disease progression [14]. In addition, circulating microRNAs have been associated with the risk of rapid progression to end-stage renal disease, further supporting their clinical relevance in risk stratification of diabetic patients [15].

These molecular findings complement current clinical guidelines, such as those from the American Diabetes Association and KDIGO, which emphasize the importance of early detection and monitoring of diabetic kidney disease based on established parameters including albuminuria and eGFR [16,17,18].

Emerging evidence further supports the utility of urinary *microRNAs* as sensitive indicators of early renal involvement and associated vascular risk in diabetes, including pediatric populations [19]. In addition, circulating *microRNAs* exist in stable extracellular forms, such as protein-bound Argonaute2 complexes, which protect them from degradation and enhance their reliability as minimally invasive biomarkers in human plasma [20]. In addition, circulating *microRNAs* are known to exist in stable extracellular forms, including protein-bound complexes such as *Argonaute2*, which protect them from degradation and contribute to their detectability and reliability as minimally invasive biomarkers in human plasma [20].

The present study aimed to evaluate serum and urinary levels of *miRNA-770-5p* in patients with T2DM across different stages of diabetic kidney disease and to assess its potential diagnostic value in relation to established clinical indicators of renal function. We hypothesized that circulating and urinary levels of hsa-miRNA-770-5p exhibit stage-dependent alterations that may serve as a non-invasive biomarker for diabetic nephropathy.

## 2. Materials and Methods

### 2.1. Study Groups

A total of 257 participants were enrolled and divided into four groups according to the presence of type 2 diabetes mellitus (T2DM), diabetic nephropathy (DN), and stage of renal function. The group of patients with T2DM and DN in CKD stages I–II included 67 patients (24 women and 43 men; mean age 64.14 ± 10.96 years). Patients with T2DM and DN who had reached end-stage renal failure and were undergoing maintenance hemodialysis (MHD) comprised 107 individuals (34 women and 73 men; mean age 63.39 ± 10.71 years). The group with T2DM without clinical or laboratory evidence of nephropathy included 45 patients (22 women and 23 men; mean age 58.49 ± 13.16 years). The control group consisted of 38 healthy individuals (32 women and 6 men; mean age 35.80 ± 9.47 years) (Table 1).

Patients with intermediate CKD stages (3a–4) were not included because the study was designed to compare early DN (CKD stages I–II) with end-stage renal disease requiring hemodialysis. These intermediate stages were not consistently available during recruitment, and their exclusion ensured group homogeneity and robust statistical comparisons. Albuminuria and proteinuria measurements were not available for all participants and were therefore not used as primary criteria for group classification. Instead, classification was based on clinical diagnosis and renal function assessment. The absence of complete albuminuria and proteinuria data is acknowledged as a limitation.

To minimize the impact of urinary dilution, all urinary biomarker levels were normalized to urine creatinine concentration and expressed as a ratio.

Because the healthy control group was younger than the patient groups, age differences were considered during statistical analysis and adjustments were performed where appropriate to account for potential confounding effects on *miRNA* levels.

### 2.2. RNA Isolation and Quantitative PCR

Blood and urine samples were collected in the morning after overnight fasting. Serum samples were obtained by centrifugation at 3000 rpm for 10 min within 2 h of collection and were subsequently stored at −80 °C until analysis. Urine samples were collected from all participants who were able to provide urine specimens, including patients undergoing maintenance hemodialysis. Hemolyzed samples were excluded from analysis.

Total RNA, including *microRNAs*, was extracted from 200 μL of serum and 1 mL of urine using a commercially available *miRNA* isolation kit according to the manufacturer’s instructions. An exogenous spike-in control was added to urine samples to control for extraction efficiency. Complementary *DNA* (*cDNA*) synthesis was performed using stem-loop primers specific for *hsa-miRNA-770-5p*.

Quantitative real-time PCR (qPCR) was carried out using a fluorescent probe-based detection system. An endogenous control was used for normalization of serum samples, whereas the exogenous spike-in control was used for normalization of urine samples. All reactions were performed in duplicate.

Relative levels of *miRNA-770-5p* were calculated using the ΔΔCt method. Briefly, Ct values of each sample were normalized to the appropriate control (endogenous control for serum and exogenous spike-in control for urine) to obtain ΔCt values. The ΔCt values of experimental samples were then compared with the mean ΔCt of the healthy control group to calculate ΔΔCt. Relative fold changes in *miRNA-770-5p* levels were calculated as 2^−ΔΔCt^.

### 2.3. Statistical Analysis

Statistical analyses were performed using SPSS software (Ver.26, IBM Corp., Armonk, NY, USA). Continuous variables are presented as mean ± standard deviation (SD) or median with interquartile range depending on data distribution. Normality was assessed using the Shapiro–Wilk test.

Age and sex were recorded for all participants. Differences between groups were assessed using appropriate statistical tests (Student’s *t*-test or ANOVA for continuous variables and chi-square test for categorical variables). Age and sex were considered as potential confounding factors in the interpretation of the results.

Comparisons among multiple groups were performed using one-way analysis of variance (ANOVA) with post hoc tests or Kruskal–Wallis tests when appropriate. Differences between two groups were evaluated using Student’s *t*-test or the Mann–Whitney U test. Statistical significance was defined as *p* < 0.05.

Receiver operating characteristic (ROC) curve analysis was performed to evaluate the ability of *miRNA-770-5p* to discriminate between study groups. A combined model including *miRNA-770-5p* and eGFR was constructed for exploratory purposes, and its discriminatory performance was assessed.

### 2.4. ROC Curve Analysis

To evaluate the diagnostic performance of *miRNA-770-5p* levels in distinguishing patients with DN from patients with T2DM without nephropathy, receiver operating characteristic (ROC) curves were constructed. The area under the curve (AUC) with 95% confidence intervals was calculated. Optimal cut-off values, sensitivity, and specificity were determined. ROC analyses were performed separately for serum and urine miRNA measurements.

## 3. Results

### 3.1. Serum Levels of miRNA-770-5p

**Figure 1:**
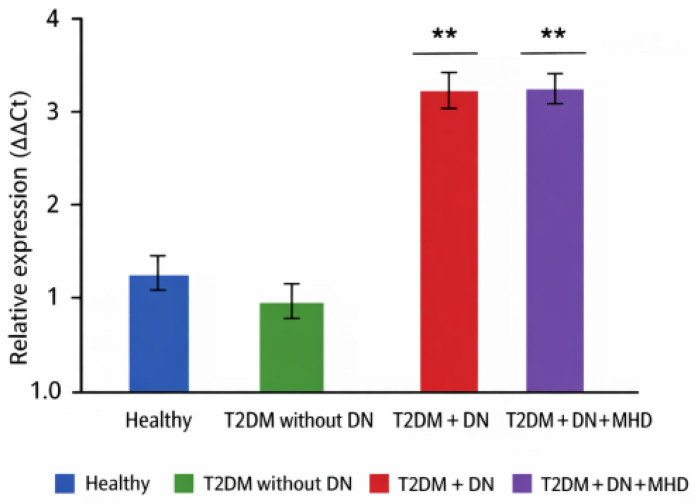
Serum *hsa-miRNA-770-5p* relative expression levels in healthy controls, patients with type 2 diabetes mellitus (T2DM) without diabetic nephropaty (DN) patients with T2DM and DN (CKD stages I–II), and patients with T2DM, DN and on maintenance hemodialysis (MHD). Relative expression was quantified using ∆∆Ct method and is presented as mean ± SD. Blue bars represent healthy controls, green bars represent T2DM without DN, red bars present T2DM with DN, and purple bars represent T2DM with DN on MHD. Statistical significance is indicated as ** *p* < 0.01 compared to the control group.

Significant differences in serum miRNA-770-5p levels were observed among the study groups (ANOVA, *p* < 0.01) (Table 2, Figure 1). Patients with T2DM without nephropathy showed slightly lower serum levels compared with healthy controls, whereas patients with T2DM and diabetic nephropathy (CKD I–II) exhibited a marked increase, approximately 3–4 times higher than controls (*p* < 0.01). Patients undergoing maintenance hemodialysis also showed elevated serum levels, which were not significantly different from those observed in patients with DN.

Patients on chronic hemodialysis also showed elevated serum levels, which were not significantly different from those in patients with DN (Figure 1).

Correlations with clinical markers:

- Negative correlation with eGFR in DN patients (r = −0.52, *p* < 0.001).
- Positive correlation with proteinuria/albuminuria (r = 0.46, *p* < 0.01).

**Figure 3:**
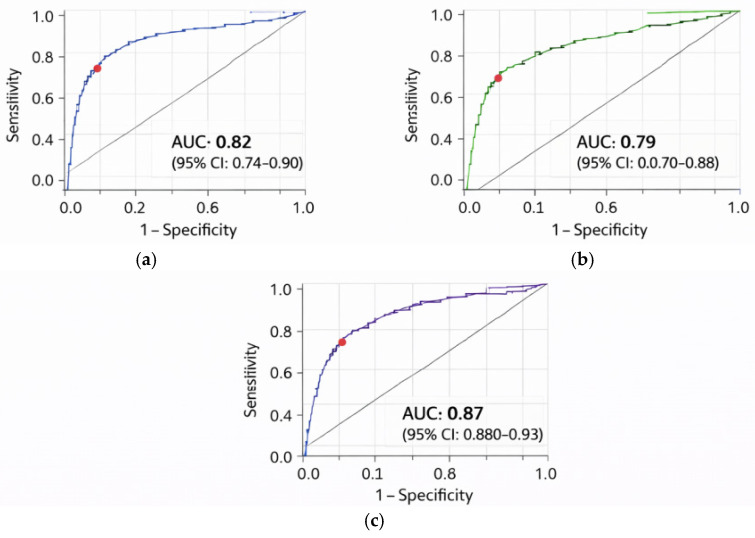
Receiver operating characteristic (ROC) curves illustrating the diagnostic performance of *hsa-miR-770-5p* for discrimination between patients with DN)and T2DM patients without DN. (**a**) ROC curve of serum *hsa-miR-770-5p*, with an area under the curve (AUC) of 0.82 (95% CI: 0.74–0.90). (**b**) ROC curve of urinary *hsa-miR-770-5p*, with an AUC of 0.79 (95% CI: 0.70–0.88). (**c**) ROC curve of a combined model including serum *hsa-miR-770-5p* and eGFR, demonstrating improved diagnostic performance (AUC = 0.87, 95% CI: 0.88–0.93). The diagonal line represents the line of no discrimination (AUC = 0.5, 95% CI: 0.48–0.54). (**a**) Serum *miRNA-770-5p* (AUC = 0.82, 95% CI: 0.78–0.85). (**b**) Urinary *miRNA-770-5p* (AUC = 0.79, 95% CI: 0.76–0.81). (**c**) Combined serum *miRNA-770-5p* + eGFR (AUC = 0.87, 95% CI: 0.86–0.92,exploratory).

ROC analysis:

Serum miRNA-770-5p discriminated DN patients from T2DM patients without nephropathy with:

- **AUC = 0.82** (95% CI: 0.74–0.90, *p* < 0.001).
- Sensitivity **78%.**
- Specificity **75%** (Figure 3a).

### 3.2. Urinary Levels of miRNA-770-5p

**Figure 2:**
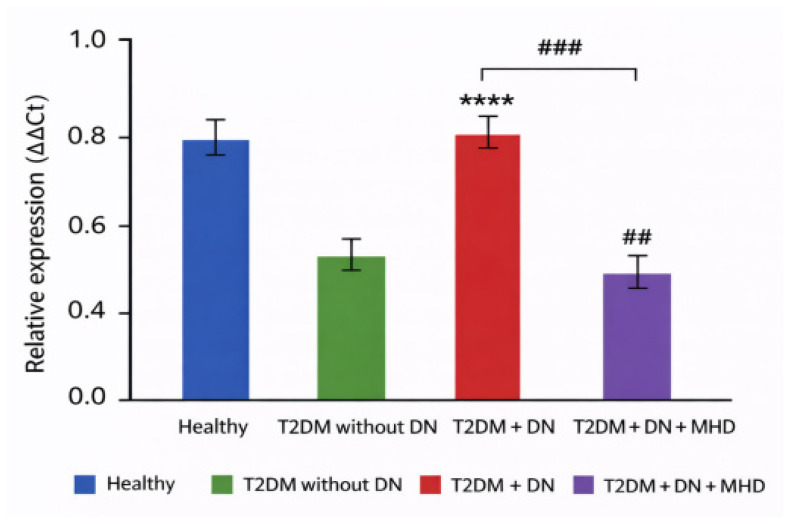
Urinary hsa-miR-770-5p relative expression levels in healthy controls, patients with type 2 diabetes mellitus (T2DM) without diabetic nephropathy (DN), patients with T2DM and DN (CKD stages I–II), and patients with T2DM, DN, and on maintenance hemodialysis (MHD). Relative expression was quantified using the ΔΔCt method and is presented as mean ± SD. Blue bars represent healthy controls, green bars represent T2DM without DN, red bars represent T2DM with DN, and purple bars represent T2DM with DN on MHD. Statistical significance is indicated as follows: **** *p* < 0.0001 vs. healthy controls; ## *p* < 0.01 and ### *p* < 0.001 between indicated groups.

Urinary *miRNA-770-5p* levels were significantly reduced in patients with DN (*p* < 0.001) (Table 3, Figure 2). A moderate decrease was observed in patients undergoing hemodialysis, although this reduction was less pronounced than in the DN group.

Correlations with clinical markers:

- Positive correlation with eGFR (r = 0.48, *p* < 0.001).
- Negative correlation with proteinuria/albuminuria (r = −0.44, *p* < 0.01).

ROC analysis:

Urinary *miRNA-770-5p* discriminated DN patients from T2DM patients without nephropathy with:

- **AUC = 0.79** (95% CI: 0.70–0.88, *p* < 0.001).
- Sensitivity **72%.**
- Specificity **76%** (Figure 3b).

### 3.3. Exploratory Combined Analysis (Serum miRNA-770-5p + eGFR)

An exploratory model combining serum *miRNA-770-5p* levels with eGFR showed improved diagnostic discrimination, with **AUC = 0.87 (95% CI: 0.80–0.93)** (Figure 3c).

This analysis should be interpreted cautiously because eGFR is part of the standard clinical assessment of diabetic nephropathy and may therefore represent a potential confounding variable.

### 3.4. Integrated Analysis of miRNA-770-5p in Serum and Urine

**Figure 4:**
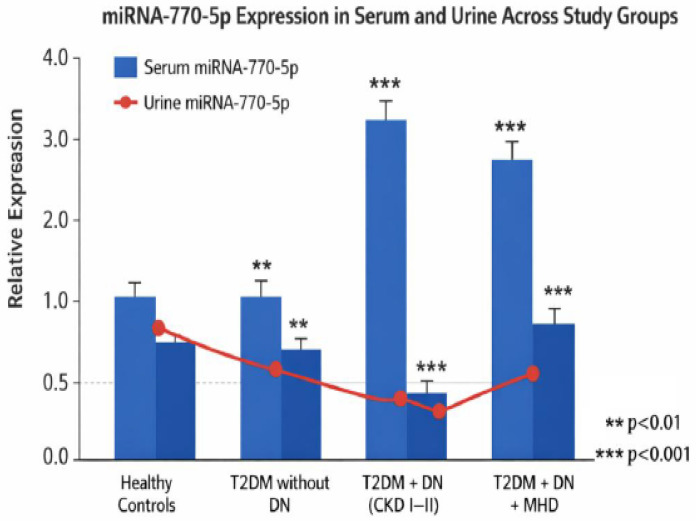
Integrated visualization of serum and urinary *hsa-miR-770-5p* expression across study groups, including healthy controls, patients with type 2 diabetes mellitus (T2DM) without diabetic nephropathy (DN), patients with T2DM and DN (CKD stages I–II), and patients with T2DM, DN, and on maintenance hemodialysis (MHD). Relative expression was quantified using the ΔΔCt method and is presented as mean ± SD. Light blue bars represent serum *hsa-miR-770-5p* levels, while dark blue bars represent urinary *hsa-miR-770-5p* levels. The red line illustrates the trend of urinary *hsa-miR-770-5p* expression across study groups. Urinary expression is presented both as bar values and as a connected line to highlight its trend across disease progression. Statistical significance is indicated as follows: ** *p* < 0.01, *** *p* < 0.001 compared to healthy controls.

An integrated overview of serum and urinary *miRNA-770-5p* demonstrates complementary patterns across study groups. Serum levels increased with disease severity, whereas urinary levels decreased as renal function deteriorated (Figure 4). Correlations with eGFR and proteinuria/albuminuria indicate that these alterations reflect both structural and functional kidney changes associated with diabetic nephropathy.

- **Scheme *770***. *p* increases with disease severity.
- Urinary *miRNA-770-5p* decreases as renal function worsens.
- Correlations with eGFR and proteinuria/albuminuria confirm that these changes reflect both structural and functional kidney alterations.

## 4. Discussion

In the present study, we observed distinct patterns of *hsa-miRNA-770-5p* expression in the serum and urine of patients with T2DM, which were associated with the presence and severity of DN. Serum levels were elevated in patients with DN and in those undergoing maintenance hemodialysis, whereas urinary levels were reduced in advanced renal disease. These complementary changes suggest that *miRNA-770-5p* may be associated with both systemic and renal-specific alterations in diabetic kidney disease and may represent a potential non-invasive biomarker.

Interestingly, serum levels of *miRNA-770-5p* in patients with T2DM without nephropathy were slightly lower than those observed in healthy controls. This observation suggests that increased circulating *miRNA-770-5p* may be associated more specifically with nephropathy rather than diabetes itself and may reflect different aspects of kidney-related pathophysiology.

The observed associations between *miRNA-770-5p* levels and established clinical markers of renal function, including eGFR and proteinuria/albuminuria, further support the potential clinical relevance of this *microRNA* in DN. Although complete albuminuria and proteinuria data were not available for all participants, the correlations identified in our cohort suggest that *miRNA-770-5p* may provide additional information complementary to conventional clinical indicators used in the evaluation of DN.

Our findings are consistent with previous studies investigating circulating and urinary *miRNAs* in diabetic nephropathy. Several reports have demonstrated dysregulation of *miRNAs* such as *miR-21*, *miR-29*, and *miR-192* in DN patients, with these *miRNAs* being associated with renal fibrosis, podocyte injury, and progressive decline in renal function. Compared with these markers, *miRNA-770-5p* appears to exhibit a distinct dual pattern characterized by elevated circulating levels and reduced urinary levels, which may provide additional insights into both systemic metabolic stress and renal-specific pathophysiological processes.

Mechanistically, *miRNA-770-5p* has been implicated in apoptosis regulation and podocyte injury through targeting TP53-regulated inhibitor of apoptosis 1 (TRIAP1) and related stress-response pathways. Podocyte loss is a key early event in DN, leading to proteinuria and glomerulosclerosis. Elevated circulating levels of *miRNA-770-5p* may reflect systemic metabolic stress and ongoing renal injury, whereas reduced urinary levels may be associated with impaired renal excretion, altered tubular handling, or intrarenal dysregulation of microRNA processing.

The exploratory analysis combining serum *miRNA-770-5p* with eGFR showed improved discrimination between DN patients and those without nephropathy. However, this finding should be interpreted with caution, as eGFR is closely related to the definition and severity of diabetic nephropathy and may therefore act as a confounding factor. Consequently, the combined model should be considered exploratory, and further studies are needed to evaluate whether *miRNA-770-5p* provides additional value beyond established clinical parameters. A limitation of this study is the difference in age and sex distribution between groups. The control group was younger, as healthy volunteers were more readily recruited from a younger population, and the proportion of females was higher in the control group. These differences may represent potential confounding factors and could have influenced the observed results. Future studies with age- and sex-matched cohorts are warranted.

In addition, the cross-sectional design of the study does not allow conclusions regarding causality or temporal relationships. Therefore, the findings should be interpreted as associations rather than evidence of predictive or diagnostic performance.

The exclusion of intermediate CKD stages (3a–4) was intentional to ensure group homogeneity and to allow clearer comparisons between early and advanced disease stages. However, future studies including the full spectrum of CKD stages will be important to better understand the dynamics of *miRNA-770-5p* across disease progression.

Overall, our results suggest that *hsa-miRNA-770-5p* is associated with disease severity in T2DM-related kidney disease and may represent a potential non-invasive biomarker that complements established clinical indicators while also providing insights into underlying pathophysiological mechanisms.

These findings may contribute to the development of novel biomarker-based strategies for early detection and monitoring of diabetic kidney disease.

### Limitations

The present study has several limitations that should be acknowledged. First, the cross-sectional design does not allow conclusions regarding causal relationships between *miRNA-770-5p* levels and the progression of diabetic nephropathy. Longitudinal studies would be necessary to determine whether alterations in circulating or urinary miRNA-770-5p levels precede the development of renal dysfunction or predict disease progression.

Second, although significant correlations between *miRNA-770-5p* levels and renal function parameters such as eGFR and proteinuria/albuminuria were observed, these clinical variables may act as potential confounding factors in the interpretation of the results. Future studies including multivariate analyses and larger patient cohorts would be useful to further clarify the independent diagnostic or prognostic value of *miRNA-770-5p.*

Third, several additional clinical variables that may influence *miRNA* levels were not systematically analyzed in the present study. Factors such as blood pressure, sex distribution, medication use, and metabolic control may affect circulating *microRNA* profiles and could potentially contribute to variability in the observed results.

Another limitation is the absence of a comparative group of patients with non-diabetic chronic kidney disease. Including such a group in future studies would help determine whether the observed alterations in *miRNA-770-5p* are specific to diabetic nephropathy or represent a more general marker of renal injury.

Finally, intermediate stages of chronic kidney disease (CKD stages 3a–4) were not included in the present study in order to maintain clear group stratification between early diabetic nephropathy (CKD I–II) and advanced renal failure requiring hemodialysis. Future investigations including patients across the full spectrum of CKD stages would allow a more detailed evaluation of *miRNA-770-5p* dynamics during disease progression.

A limitation of this study is the difference in age and sex distribution between groups. The control group was younger, as healthy volunteers were more readily recruited from a younger population. In addition, the proportion of females was higher in the control group compared to the patient groups. These differences may represent potential confounding factors and could have influenced the observed results. Therefore, the findings should be interpreted with caution. Future studies with age- and sex-matched cohorts are warranted to confirm these findings.

## 5. Conclusions

*hsa-miRNA-770-5p* demonstrates disease-severity-dependent changes in serum and urine of patients with T2DM, showing elevated serum levels and decreased urinary levels in DN. These patterns correlate with eGFR and proteinuria/albuminuria, supporting both diagnostic and mechanistic relevance. The dual-pattern expression distinguishes early renal injury from systemic diabetes effects, highlighting *miRNA-770-5p* as a promising non-invasive biomarker that could complement established clinical markers and aid in early detection, monitoring, and understanding of diabetic nephropathy. Future studies including intermediate CKD stages (3a–4) are warranted to assess its utility across the full spectrum of DN progression.

hsa-miRNA-770-5p demonstrates disease-severity-dependent changes in serum and urine in patients with type 2 diabetes mellitus, with increased serum levels and decreased urinary levels in diabetic nephropathy. These expression patterns correlate with eGFR and proteinuria/albuminuria, supporting both their diagnostic and pathophysiological relevance.

The observed dual-pattern expression may help distinguish renal injury from systemic diabetic effects, highlighting miRNA-770-5p as a promising non-invasive biomarker that could complement established clinical parameters in the detection and monitoring of diabetic kidney disease.

Further studies, including intermediate CKD stages (3a–4), are warranted to evaluate its clinical utility across the full spectrum of disease progression.

## Figures and Tables

**Table 1:** Demographic characteristics of the study groups (mean ± SD).

Group	n	Age (Years, Mean ± SD)	Female	Male
T2DM + DN (CKD I–II)	67	64.14 ± 10.96	24	43
T2DM + DN + MHD	107	63.39 ± 10.71	34	73
T2DM without DN	45	58.49 ± 13.16	22	23
Healthy controls	38	35.80 ± 9.47	32	6
Total	257	—	112	145

T2DM—type 2 diabetes mellitus; DN—diabetic nephropathy; MHD—maintenance hemodialysis; CKD—chronic kidney disease.

**Table 2:** Serum miRNA-770-5p relative levels (mean ± SD).

Group	n	Mean ± SD	Fold Change vs. Controls
Healthy controls	38	1.00 ± 0.12	1.0
T2DM without DN	45	0.75 ± 0.09	0.75
T2DM + DN (CKD I–II)	67	3.12 ± 0.22	3.1
T2DM + DN + MHD	107	3.05 ± 0.25	3.0

**Table 3:** Urinary *miRNA-770-5p* relative levels (mean ± SD).

Group	n	Mean ± SD	Fold Change vs. Controls
Healthy controls	38	1.00 ± 0.11	1.0
T2DM without DN	45	0.85 ± 0.08	0.85
T2DM + DN (CKD I–II)	67	0.12 ± 0.02	0.12
T2DM + DN + MHD	107	0.30 ± 0.04	0.30

## Data Availability

The original contributions presented in this study are included in the article. Further inquiries can be directed to the corresponding author.

## Abbreviations

**Abbreviation**	**Full term**
T2DM	Type 2 diabetes mellitus
DN	Diabetic nephropathy
MHD	Maintenance hemodialysis
CKD	Chronic kidney disease
eGFR	Estimated glomerular filtration rate
miRNA	MicroRNA
ROC	Receiver operating characteristic
AUC	Area under the curve
ΔΔCt	Delta-delta cycle threshold (method for relative gene expression)
qPCR	Quantitative real-time polymerase chain reaction
